# Sex-Based Differences in Diabetes Prevalence and Risk Factors: A Population-Based Cross-Sectional Study Among Low-Income Adults in China

**DOI:** 10.3389/fendo.2019.00658

**Published:** 2019-09-25

**Authors:** Hongyan Zhang, Jingxian Ni, Changshen Yu, Yanan Wu, Jingyan Li, Jie Liu, Jun Tu, Xianjia Ning, Qing He, Jinghua Wang

**Affiliations:** ^1^Department of Endocrinology and Metabolism, Tianjin Medical University General Hospital, Tianjin, China; ^2^Department of Neurology, Tianjin Medical University General Hospital, Tianjin, China; ^3^Department of Epidemiology, Tianjin Neurological Institute, Tianjin, China; ^4^Key Laboratory of Post-Neuroinjury Neuro-Repair and Regeneration in Central Nervous System, Ministry of Education and Tianjin City, Tianjin Neurological Institute, Tianjin, China; ^5^Department of Neurology, Tianjin Huanhu Hospital, Tianjin, China

**Keywords:** sex differences, diabetes mellitus, prevalence, risk factors, epidemiology

## Abstract

**Aims:** The prevalence of diabetes mellitus (DM) among adults has reached epidemic proportions worldwide, including China. In China, sex-based differences in the prevalence and risk factors of DM may exist, particularly among low-income individuals. Thus, we assessed these differences in the prevalence of DM and its risk factors in a low-income Chinese population.

**Materials and Methods:** Residents aged ≥45 years without histories of strokes or cardiovascular disease were recruited for this study. Multivariate logistic regression analyses were performed to assess the association of risk factors with DM prevalence.

**Results:** This study included 3,725 participants (41.2%, men; 58.8%, women). The mean age of the women (61.12 years) was higher than that of the men (59.14 years, *P* < 0.001). There was no significant sex-based difference in DM prevalence (men, 14.1%; women, 14.5%). Overweight, obesity, high triglyceride levels, and hypertension were independent risk factors for DM in both sexes. However, high-density lipoprotein-cholesterol levels were negatively associated with DM risk among men [odds ratio (OR), 0.544; 95% confidence interval (CI), 0.355–0.833; *P* = 0.005]. Among women, advanced age and high low-density lipoprotein-cholesterol levels were independent risk factors for DM; there was a higher DM risk for women aged 55–74 years than for those aged 45–54 years; however, physical activity was associated with an increased risk of DM (OR, 1.705; 95% CI, 1.195–2.432; *P* = 0.003).

**Conclusions:** These findings suggest a crucial need to implement individualized blood pressure, weight, and lipid managements in low-income populations in China to reduce the burden of DM, especially among older women.

## Introduction

Diabetes mellitus (DM) is rapidly becoming one of the most common non-communicable diseases worldwide (1), and its prevalence among adults has increased over the past few decades (2). The International Diabetes Federation estimated that 382 million people, worldwide, had DM in 2013; this number is expected to rise to 592 million by 2035, including an increase from 98.4 to 142.7 million people in China (3). The current estimate of the number of adults (aged >20 years) in China with DM is 92.4 million (including 60.7% of undiagnosed cases) (4). Between 2002 and 2020, the DM age-standardized mortality is projected to rise 1.1% among men and 1.3% among women. In 2002, DM was ranked as the eleventh leading cause of death, globally; it is projected to be seventh by 2030 (5). Additionally, DM is also a major cause of cardiovascular disease (CVD), chronic kidney disease, blindness, and amputations. As a result, between 2006 and 2015, the national income of China was estimated to have declined by $558 billion due to cardio- and cerebrovascular diseases and DM (6).

Numerous studies have demonstrated that conventional CVD risk factors, including age, sex, body mass index (BMI), blood pressure (BP), and dyslipidemia, are also associated with developing DM (4, 7–10). Sex-related differences in DM prevalence and associated risk factors were previously established (11–13). However, few studies have reported on sex-related differences in China, especially among low-income, poorly educated people.

In China, approximately half of the population lives in rural areas where incomes and educational attainment are low. These factors play decisive roles in increasing the burden of disease; thus, we assessed sex-related differences in DM prevalence and associated risk factors in a low-income population in rural China.

## Patients and Methods

### Study Population

The baseline investigation for this population-based cohort study was conducted between April 2014 and January 2015, as previously described (14–16). Briefly, the total study population included 14,251 individuals from 18 administrative villages. Approximately 95% of the participants were low-income farmers, with a 2014 per capita disposable income of <1,600 USD (17). All residents aged ≥45 years, without diagnosed CVD, were recruited into this study.

The study was approved by the medical research ethics committee at Tianjin Medical University General Hospital; written informed consent was obtained from each participant during recruitment.

### Data Collection and Risk Factor Definitions

Pre-designed questionnaire surveys were administered, in person, by trained epidemiology researchers. Demographic information, e.g., name, sex, date of birth, and educational level, were obtained from existing records.

Individual and family medical histories, including hypertension, DM, stroke, transient ischemic attacks, and coronary heart disease, were obtained from medical records or according to participant self-reports.

Lifestyle information included cigarette smoking and alcohol consumption habits as well as physical activity levels and self-reported salt intake. Cigarette smoking was defined as smoking >1 cigarette/day for ≥1 year; participants were categorized as non-smokers, former smokers (stopped smoking for at least 6 months), and current smokers. Alcohol consumption was defined as drinking >500 g (equivalent to 17 oz) of alcohol/week for >1 year; participants were categorized as non-drinkers, former drinkers (temperance for ≥6 months), and current drinkers, both for men and for women. Physical activity was defined as being involved in a physical activity ≥5 days/week for ≥30 min/day. Self-reported salt intake was defined as mild, moderate, and according to self-reported salt intake; mild was defined as with the salt intake of <6 g/day, moderate was defined as with the salt intake of 6–12 g/day, and heavy was defined as with the salt intake of >12 g/day.

### Physical Examinations and Measurements

Physical examinations, e.g., those of BP [systolic BP (SBP) and diastolic BP (DBP)], height, and weight, were performed at the local village clinic during the survey. The levels of fasting plasma glucose (FPG), total cholesterol (TC), triglycerides (TG), high-density lipoprotein cholesterol (HDL-C), and low-density lipoprotein cholesterol (LDL-C) were measured at the Ji County People's Hospital. Body mass index (BMI) was calculated as weight (kg) divided by the square of height (m^2^).

Hypertension was defined as SBP ≥140 mmHg, DBP ≥90 mmHg, or taking hypertension medications. DM was defined as an FPG level ≥7.0 mmol/L, a previous history of diagnosed diabetes, or having a prescription for insulin or oral antidiabetic drugs (18). Weight classifications were based on BMI (normal, 18.5–23.9 kg/m^2^; low-weight, <18.5 kg/m^2^; overweight, 24.0–27.9 kg/m^2^; obesity, ≥28.0 kg/m^2^) (19). Elevated blood lipids were defined as TC ≥ 6.22 mmol/L, TG ≥ 2.26 mmol/L, and LDL-C ≥ 4.14 mmol/L; low HDL-C was defined as <1.04 mmol/L (20).

### Statistical Analyses

The participants were categorized into four age groups: 45–54, 55–64, 65–74, and ≥75 years. Educational levels were categorized into three groups: illiterate (no formal education), 1–6 years of education, and >6 years of education. Continuous variables are presented as means and standard deviations, with comparisons between two groups being conducted using Student's *t*-tests. Categorical variables are presented as numbers and frequencies; between-group comparisons were performed using chi-squared tests. Multiple linear regression analyses were used to evaluate the association of DM with factors showing statistical significance in the univariate analyses. The relationships are presented as odds ratios (ORs) with 95% confidence intervals (CIs); two-tailed *P* < 0.05 were considered statistically significant. SPSS for Windows (version 19.0; SPSS, Chicago, IL, USA) was used for the analyses.

## Results

The participant selection process was previously described (14). Briefly, 4,012 of the 5,380 qualified residents were interviewed during the study period, yielding a 75% response rate. After excluding 223 residents with prior histories of CVD or strokes and 64 without FPG measurements, 3,725 individuals were ultimately included in the analyses.

### Demographic Characteristics

In this study, males (mean age, 61.12 years) accounted for 41.2% (*n* = 1,536) of the participants; females (mean age, 59.14 years) accounted for 58.8% (*n* = 2,189) of the participants and were significantly younger than the men (*P* < 0.001). The men had a higher prevalence of hypertension, higher mean SBP and DBP values, and higher HDL-C levels (*P* < 0.05) than did the women. However, the frequencies of illiteracy and obesity were higher among women than among men; women were more likely to have elevated levels of TC, TG, and LDL-C (*P* < 0.05; Table 1).

**Table 1:** Demographical characteristics for all participants in this study by gender.

**Groups**	**Men**	**Women**	***P***
Participants, *n* (%)	1,536 (41.2)	2,189 (58.8)	–
Age, means (SD), years	61.12 (9.91)	59.14 (9.43)	<0.001
Age group, *n* (%)			<0.001
45 54 years	424 (27.6)	780 (35.6)	
55 64 years	622 (40.5)	873 (39.9)	
65 74 years	332 (21.6)	384 (17.5)	
≥75 years	158 (10.3)	152 (6.9)	
Education, means (SD), years	6.41 (3.22)	4.82 (3.61)	<0.001
Education, *n* (%)			<0.001
0 years	134 (8.7)	516 (23.6)	
1 6 years	691 (45.0)	975 (44.5)	
>6 years	711 (46.3)	698 (31.9)	
Smoking status, *n* (%)			<0.001
Never smoking	654 (42.6)	2,136 (97.6)	
Ever smoking	164 (10.7)	7 (0.3)	
Current smoking	718 (46.7)	46 (2.1)	
Alcohol consumption, *n* (%)			<0.001
Never drinking	984 (64.1)	2,159 (98.6)	
Ever drinking	45 (2.9)	1 (0)	
Current drinking	507 (33.0)	29 (1.3)	
Physical activity, *n* (%):			0.893
Yes	153 (10.0)	221 (10.1)	
No	1,383 (90.0)	1,968 (89.9)	
Intake salt, *n* (%):			0.584
Mild	253 (16.5)	377 (17.2)	
Moderate	1,014 (66.0)	1,456 (66.5)	
Heavy	269 (17.5)	356 (16.3)	
Hypertension, *n* (%)	1,094 (71.2)	1,455 (66.5)	0.002
SBP, means (SD), mmHg	147.68 (21.35)	145.69 (22.59)	0.006
DBP, means (SD), mmHg	88.50 (11.18)	85.67 (11.36)	<0.001
BMI, means (SD), years	25.20 (3.46)	25.83 (3.80)	<0.001
BMI group, *n* (%)			<0.001
Low-weight	27 (1.8)	38 (1.7)	
Normal weight	551 (35.9)	659 (30.1)	
Over-weight	637 (41.5)	935 (42.7)	
Obesity	321 (20.9)	557 (25.4)	
FPG, means (SD), mmol/L	5.91 (1.42)	5.93 (1.66)	0.660
FPG ≥ 7.0 mmol/L	184 (12.0)	259 (11.8)	0.891
TC, means (SD), mmol/L	4.62 (1.00)	5.04 (1.11)	<0.001
TC≥ 6.22 mmol/L	99 (6.4)	290 (13.2)	<0.001
TG, means (SD), mmol/L	1.61 (1.24)	1.87 (1.22)	<0.001
TG≥ 2.26 mmol/L	273 (17.8)	543 (24.8)	<0.001
HDL-C, means (SD), mmol/L	1.39 (0.43)	1.50 (0.48)	<0.001
HDL-C ≤ 1.04 mmol/L	294 (19.1)	252 (11.5)	<0.001
LDL-C, means (SD), mmol/L	2.61 (1.20)	2.76 (1.28)	<0.001
LDL-C≥ 4.14 mmol/L	101 (6.6)	195 (8.9)	0.010

*SBP, systolic blood pressure; DBP, diastolic blood pressure; BMI, body mass index; TC, total cholesterol; TG, triglycerides; HDL-C, high density lipoprotein cholesterol; LDL-C, low density lipoprotein cholesterol*.

### Prevalence of DM

Table 2 displays the prevalence of DM among men and women in this study; the overall DM prevalence was 14.3%. Among men, the prevalence of DM increased with increasing BMI; the prevalence of DM was significantly higher among individuals with hypertension than among those with normal blood pressure. Moreover, the prevalence of DM among women increased with increasing age, education level, and BMI. There was a higher prevalence of DM among women with hypertension and those who were physically active.

**Table 2:** The prevalence of DM for all participants in this study by gender.

**Groups**	**Men**		**Women**	
	**Prevalence**	***P***	**Prevalence**	***P***
Participants, *n* (%)	216 (14.1)	–	317 (14.5)	–
Age group, *n* (%)		0.815		<0.001
45 54 years	63 (14.9)		66 (8.5)	
55 64 years	81 (13.0)		137 (15.7)	
65 74 years	49 (14.8)		87 (22.7)	
≥75 years	23 (14.6)		27 (17.8)	
Education, *n* (%)		0.277		<0.001
0 years	14 (10.4)		99 (19.2)	
1 6 years	106 (15.3)		147 (15.1)	
> 6 years	96 (13.5)		71 (10.2)	
Smoking status, *n* (%)		0.103		0.166
Never smoking	101 (15.4)		306 (14.3)	
Ever smoking	26 (15.9)		1 (14.3)	
Current smoking	89 (12.4)		10 (21.7)	
Alcohol consumption, *n* (%)		0.311		0.886
Never drinking	143 (14.5)		313 (14.5)	
Ever drinking	10 (22.2)		0	
Current drinking	63 (12.4)		4 (13.8)	
Physical activity, *n* (%):		0.179		0.001
Yes	27 (17.6)		49 (22.2)	
No	189 (13.7)		268 (13.6)	
Intake salt, *n* (%):		0.975		0.415
Mild	38 (15.0)		60 (15.9)	
Moderate	138 (13.6)		218 (15.0)	
Heavy	40 (14.9)		49 (13.8)	
Hypertension, *n* (%)		<0.001		<0.001
Yes	182 (16.6)		263 (18.1)	
No	34 (7.7)		54 (7.4)	
BMI group, *n* (%)		<0.001		<0.001
Normal weight	46 (8.0)		65 (9.3)	
Over-weight	96 (15.1)		150 (16.0)	
Obesity	74 (23.1)		102 (18.3)	

*SBP, systolic blood pressure; DBP, diastolic blood pressure; BMI, body mass index; P-value indicated the statistical significance to compare the differences of the prevalence between two groups or P for trend for more than two groups*.

Table 3 shows that men with DM had higher TG levels and lower HDL-C levels than did men without DM. Moreover, the levels of TC, TG, and LDL-C were significant higher among women with DM than among those without DM, but a converse trend was found for HDL-C levels.

**Table 3:** Differences of the lipids levels among individuals with or without DM for all participants in this study by gender.

**Groups**	**Men**		**Women**	
	**Means (*SD*)**	***P***	**Means (*SD*)**	***P***
TC, mmol/L		0.142		0.001
DM	4.73 (1.18)		5.22 (1.19)	
Non-DM	4.60 (0.97)		5.01 (1.09)	
TG, mmol/L		<0.001		<0.001
DM	1.97 (1.31)		2.23 (1.64)	
Non-DM	1.55 (1.22)		1.81 (1.13)	
HDL-C, mmol/L		<0.001		0.035
DM	1.25 (0.37)		1.45 (0.54)	
Non-DM	1.41 (0.43)		1.51 (0.46)	
LDL-C, mmol/L		0.076		0.002
DM	2.74 (1.34)		3.00 (1.50)	
Non-DM	2.59 (1.18)		2.72 (1.23)	

*TC, total cholesterol; TG, triglycerides; HDL-C, high density lipoprotein cholesterol; LDL-C, low density lipoprotein cholesterol. P-value indicated the statistical significance to compare the differences of the lipids levels between DM groups and non-DM groups*.

### Sex-Based Differences in DM Risk Factors

In the multivariate analysis, overweight, obesity, hypertension, and high TG levels were independent risk factors for DM in both sexes. Compared with those having normal weights, the prevalence of DM was 59.4% higher among men who were overweight (OR, 1.594; 95% CI, 1.083–2.346; *P* = 0.001) and 125.0% higher among those who were obese (OR, 2.250; 95% CI, 1.462–3.461; *P* = 0.018). The prevalence of DM was also 115.5% higher among men with hypertension (*P* < 0.001) than among those with normal BPs; the prevalence of DM among men increased 11.8% with each 1 mmol/L elevation in the TG level (OR, 1.118; 95% CI, 1.010–1.238; *P* = 0.032). However, there was a negative association between HDL-C levels and DM prevalence (OR, 0.544; 95% CI, 0.355–0.833; *P* = 0.005; Table 4).

**Table 4:** Sex-based differences in associated factors of DM in the multivariate analyses.

**Groups**	**Reference**	**Men**			**Women**		
		**OR**	**95%CI**	***P***	**OR**	**95%CI**	***P***
Age group:	45~54 years						
55~64 years		–	–	–	1.580	1.120–2.230	0.009
65~74 years		–	–	–	2.301	1.538–3.444	<0.001
≥75 years		–	–	–	1.677	0.957–2.939	0.071
Education:	>6 years	–	–	–			
0 years		–	–	–	1.362	0.923–2.009	0.119
1~6 years		–	–	–	1.130	0.806–1.583	0.478
BMI group	Normal weight						
Over-weight		1.594	1.083-2.346	0.001	1.687	1.219–2.334	0.002
Obesity		2.250	1.462ity334-3.461	0.019	1.840	1.287–2.629	0.001
Hypertension	No	2.155	1.455tensio-3.191	<0.001	1.967	1.424–2.718	<0.001
Physical activity	No	–	–	–	1.705	1.195–2.432	0.003
TC, mmol/L	–	–	–	–	0.964	0.835–1.113	0.620
TG, mmol/L	–	1.118	1.010l/L113-1.238	0.032	1.206	1.088–1.336	<0.001
HDL-C, mmol/L	–	0.544	0.355l/L336-0.833	0.005	1.002	0.743–1.352	0.988
LDL-C, mmol/L	–	–	–	–	1.114	1.002–1.237	0.045

*OR, odds ratio; CI, confidence interval; BMI, body mass index; TC, total cholesterol; TG, triglycerides; HDL-C, high density lipoprotein cholesterol; LDL-C, low density lipoprotein cholesterol*.

Among women, old age was an independent risk factor associated with DM. Using the 45 to 54-year-old group as the reference group, there were higher prevalences of DM among those aged 55–64 years (OR, 1.580; 95% CI, 1.120–2.230; *P* = 0.009) and among those aged 65–74 years (OR, 2.301; 95% CI, 1.538–3.444; *P* < 0.001); this relationship disappeared for women ≥75 years (OR, 1.677; 95% CI, 0.957–2.939; *P* = 0.071). Similarly, women who were overweight (OR, 1.687; 95% CI, 1.219–2.334; *P* = 0.002), obese (OR, 1.840; 95% CI, 1.287–2.629; *P* = 0.001), had hypertension (OR, 1.967; 95% CI, 1.424–2.718; *P* < 0.001), or participated in physical activities (OR, 1.705; 95% CI, 1.195–2.432; *P* = 0.003) had elevated TG (OR, 1.206; 95% CI, 1.088–1.336; *P* < 0.001) and LDL-C (OR, 1.114; 95% CI, 1.002–1.237; *P* = 0.045) levels and demonstrated a higher DM prevalence than did their reference counterparts (Table 4).

## Discussion

This is the first report describing sex-based differences in DM prevalence and its determinants in a low-income and poorly educated population in China. Overall, the observed prevalence of DM was 14.1% in men and 14.5% in women. Further, overweight, obesity, hypertension, and high TG levels were independent risk factors for DM in both sexes. Among men, a high HDL-C value was an independent protective factor for DM. Among women, advanced age, physical activity, and high LDL-C levels were additional independent risk factors of DM.

Over the last several decades, China has experienced a dramatic increase in the prevalence of DM, rising from 1% in 1980 (21) to 2.5% in 1994 (22), 2.6% in 2002 (23), 9.7% in 2008 (4), and 11.6% in 2010 (24). However, the sex-based differences in the DM prevalence have remained unknown. A recent meta-analysis showed that the pooled prevalence rates were 9.9% (95% CI, 8.8–11.0%) among men, and 11.6% (95% CI, 10.0–13.1%) among women (25). A male prevalence of DM was observed in a large nationwide survey in which the age-standardized prevalence of total DM (both previously diagnosed and undiagnosed diabetes) was 10.6% among men and 8.8% among women (4). However, other studies indicated a higher frequency of DM among women than among men when the individuals were grouped according to age, educational level, hypertension, and BMI (8). In the present study, a significant, sex-based difference in the prevalence of DM within this low-income population was not observed. Disparities in the various study designs and the criteria used to establish a DM diagnosis may partly explain these differences.

A positive association between advanced age and DM or FPG level has been confirmed in many studies (25–28). Since the 1970s, the prevalence of DM has been observed to increase rapidly with age, with an overall prevalence of 14.1% in Chinese people aged 65–74 years (25). A nationally representative, cross-sectional survey was conducted in 2013 in mainland China; 170,287 participants were included. In that study, the estimated overall prevalence of total diabetes was 10.9% (95% CI, 10.4–11.5%), including 10.2% (95% CI, 9.7–10.7%) among women and 11.7% (95% CI, 10.9–12.4%) among men (24). The prevalence of diabetes increased with age, with the prevalence in 40–49 years vs. ≥70 years of 7.7 vs. 16.7% (28), and 11.3 vs. 23.5% in China (24). Consistent with the previous studies, advanced age was independently associated with DM only among women in the present study. Compared to 45 to 54-year-old individuals, the prevalence of DM increased by 58.0% among 55 to 64-year-olds and by 130.1% among 65 to 74-year-olds. The higher risk for women and advanced age to develop DM may be attributed to sex-based differences in fat storage locations (29) and islet beta-cell dysfunction in elderly individuals (30).

Obesity is a major independent and modifiable risk factor for DM, and many epidemiological studies have suggested that the prevalence of DM is higher among obese individuals (31, 32). Further, a high BMI is a predictor of developing DM among individuals aged 65–96 years (33) and may be an important pathogenic factor associated with DM in the elderly (33–35). A prior study, involving 2,478 children and adolescents (3–18 years old), also found that a 1-standard deviation increase in BMI was associated with a 45% increase in DM risk among adults (36). In the present study, BMI was an independent risk factor associated with DM in both sexes. Compared to normal-weight individuals, the prevalence of DM increased by 59.4% among men and 68.7% among women for those who were overweight, and by 125.0 and 84%, respectively, for those who were obese. Thus, a national effort is recommended to prevent childhood obesity by improving education and social conditions in this demographic; without such interference, a significant number of these individuals will eventually develop obesity and diabetes in adulthood.

In a women's health study, the risk of developing DM was twice as high among individuals with hypertension than that among those with SBPs between 120 and 129 mmHg (4). In a cohort study, both SBP and DBP were associated with elevated risks of new-onset diabetes; the risk of developing DM increased by 58% following a 20-mmHg increase in SBP and by 52% following a 10-mmHg increase in DBP (37). However, among men, an association was not observed between baseline BP and the risk of developing DM, after adjusting for covariates (38). In the present study, hypertension was an independent risk factor for DM in both sexes, contributing to a 115.5% (men) or 96.7% (women) increase in the prevalence of DM, compared with normotensive individuals. Elevated BP may increase the risk of DM by inducing chronic inflammation and endothelial dysfunction (39–41). Thus, chronic inflammation may partly mediate the link between some risk factors (obesity and hypertension) and DM.

The relationship between hypertriglyceridemia and developing DM or an elevated FPG level was established in previous studies. Specifically, individuals with high TG, TC, and LDL-C levels and those with low HDL-C levels were more likely to develop DM (42–44). Every 10 mg/dL increase in TG level increases the risk of DM by 4% (45). In contrast, several prospective studies failed to find a relationship between TG levels and DM, after adjusting for conventional risk factors (46–48). The impact of dyslipidemia on incident DM may be mediated by the inhibition of insulin secretion or the development of insulin resistance (49, 50). In both sexes, in the present study, high TG levels were independent risk factors for DM; for each 1-mmol/L increase in the TG level, the risk of DM rose by 11.8% among men and 21.1% among women. Moreover, each 1-mmol/L increase in HDL-C level decreased the risk of DM by 46.6%, among men.

A significant inverse relationship between educational level and DM prevalence was documented in previous studies. Compared with individuals having ≥13 years of education, those with ≤6 years of education (OR, 2.10; 95% CI, 1.27–3.48) or with 7–12 years of education (OR, 1.62; 95% CI, 1.04–2.52) had higher risks of developing type 2 diabetes, after adjusting for age, sex, medical characteristics, lifestyle factors, and stress levels; the OR for women with ≤6 years of education was particularly high (OR, 10.16; 95% CI, 2.08–49.53), even after adjusting for covariates (12). However, a similar trend was not observed for men (51, 52). Similarly, an association between DM and educational level was not observed among either men or women in the current study population.

Previous studies have shown that increased physical activity and reduced calorie intake significantly decrease the incidence of type 2 DM (53–55). Physical activity, combined with dietary modifications, was recommended as an initial intervention for those with intermediate hyperglycemia and for those with type 2 DM (56). With rapid economic growth and the associated industrialization, urbanization, and lifestyle changes (high calorie, fat, sugar, and sodium diets as well as decreased physical activity), prediabetes and diabetes have reached epidemic proportions in the Chinese population. In a large, nationwide survey on the prevalence and control of DM in Chinese adults, physical activity was positively associated with a higher risk of prediabetes, but not with DM (24). Insufficient physical activity among rural people over 40 years old increases the risk of type 2 DM and metabolic syndrome (57). Inconsistent with these studies, in this cross-sectional study, physical activity was negatively associated with the risk of DM in women, but not in men. All participants in this study were from a low-income, poorly educated population; thus, poor health awareness may have reduced the physical activity initiatives before diagnosing DM. However, this is the cross-sectional study, not a cohort study; those individuals being diagnosed with DM would initiative participate in the physical activity after being diagnosed with DM. Thus, increased physical activity after diagnosed with DM may explain the negative association of physical activity with DM risk in this cross-sectional study.

Several studies have demonstrated that elevated the dietary salt intake associated with hypertension, BMI, and the risk of CVD death (58–61). However, we assessed the sex difference in association of the dietary salt intake with DM prevalence and risk factors using self-reported salt intake categories in the present study. There is no significant sex difference in DM prevalence among the different salt intake groups.

This study has several limitations. First, the study population was recruited from villages in Tianjin, China; therefore, the findings may not extend to the overall national population. Second, participants were only assessed for FPG levels; the absence of impaired glucose-tolerance testing or glycosylated hemoglobin data may have underestimated the risk of DM. Moreover, the reliance of the study on self-reported DM, in this poorly educated population, may have also underestimated the prevalence of DM. Furthermore, the self-reported salt intake in this low education population may impact the accurate evaluation of the association of salt intake with DM risk. Finally, socio-economic status and nutrition data were not collected due to the limited education of the target population, we only collected the information of dietary salt intake by categorized groups, but absence of quantitative analysis.

This report describes sex-based differences in DM prevalence and its determinants in a low-income, poorly educated population, in China. The observed prevalence of DM was 14.1% among men and 14.5% among women. Further, overweight, obesity, hypertension, and high TG levels were independent risk factors for DM in both sexes. Among men, a high HDL-C level was an independent protective factor for DM. Among women, advanced age, physical activity, and high LDL-C levels were additional independent risk factors of DM. These findings suggest a crucial need to implement individualized BP, weight, and lipid management among low-income populations, in China, to reduce the burden of DM, especially among older women.

## Ethics Statement

The study was approved by the ethics committee for medical research at Tianjin Medical University General Hospital, and a written informed consent was obtained from each participant during recruitment.

## Author Contributions

JW, XN, and QH were involved in conception, design, and data collection. HZ, JN, and CY were involved in manuscript drafting for this article. HZ, JN, CY, YW, JLi, JLiu, JT, XN, QH, and JW were involved in data collection and case diagnosis and confirmation for this article. JW and XN were involved in data analysis, data interpretation, and critical review for this article. All authors read and approved the final manuscript.

### Conflict of Interest

The authors declare that the research was conducted in the absence of any commercial or financial relationships that could be construed as a potential conflict of interest.
